# The role of institutional trust in the formalization of women’s savings and loans groups: Experiences from the Nigeria for Women Project

**DOI:** 10.12688/gatesopenres.16389.1

**Published:** 2026-06-16

**Authors:** Cody Bock, Anna McBride, Thomas de Hoop, Christopher Paek, Yetunde Fatogun, Michael Gboyega Ilesanmi

**Affiliations:** 1 American Institutes for Research, Arlington, Virginia, USA; 2 World Bank Nigeria, Abuja, Nigeria

**Keywords:** Nigeria, women’s empowerment, self-help groups, savings groups, institutional trust, formalization

## Abstract

This paper presents evidence on the role of institutional trust in the formation and formalization of government-supported women’s savings groups, known as Women Affinity Groups (WAGs) and implemented through the Nigeria for Women Project (NFWP). While existing research has shown the vital role of peer trust in the operation and effectiveness of savings groups, less is known about how institutional trust affects the formalization and scale up of savings groups. This paper examines how underlying drivers of institutional trust and mistrust shape savings group formalization processes and outcomes. In-depth interviews and focus group discussions with participants, community members, and staff of the NFWP suggest that project implementers were able to overcome initial mistrust of the NFWP by leveraging community structures, demonstrating consistency, and providing promised services. Community mobilization through reputable social networks contributed to the facilitation of group formation, which in turn helped address challenges associated with a weak institutional environment and poor experiences with previous livelihood interventions. A demonstrated record of success also enabled building the credibility of the project.

## Introduction

Development practitioners often seek to formalize and institutionalize community economic development initiatives to more easily deliver financial and institutional support ( Igalla et al., 2021), promote sustainability ( de Hoop et al., 2023; Clift, 1990), strengthen social cohesion ( Anderson & Francois, 2008), and efficiently achieve impact at scale ( Baliwada et al., 2017; Opondo et al., 2011). Particularly for economic initiatives, formalization through state institutions can lend legitimacy to development programs and provide necessary social and economic protection ( Xheneti et al., 2017).

In recent years, savings and credit groups, including village savings and loan associations and self-help groups have gained considerable traction within the development community for their potential to provide poor and marginalized populations, especially those living just above or close to the poverty line, with accessible credit ( Hendricks & Chidiac, 2011), livelihood development ( Osei-Fosu et al., 2019), and social support ( Ibarguen, 2014; Musinguzi, 2016). These voluntary groups, often comprised of a majority or only women, provide financial and social support to members while offering a platform for participants to save money and receive low-risk credit ( Desai et al., 2022). Although such groups were initially informal, national governments such as India, Kenya, Nigeria, and Uganda have promoted savings and loan groups in their countries through formal state-led programs ( Namisango et al., 2022). The formalization of savings and loan groups shows considerable promise to enable the extreme poor to overcome poverty and to make progress toward the UN Sustainable Development Goals ( Volmer, 2016).

Savings group formalization is not a uniform process and may involve registering groups with the government or an organization, providing resources (e.g., technical staff, loans, grants), instituting a training curriculum, and/or establishing fixed procedures to govern the groups ( Tafa, 2019; Volmer, 2016). Though formalization can enable development actors to more efficiently implement, support, and scale-up savings and loan groups, the process introduces a new institutional or organizational actor into these traditionally informal, community-based structures ( Baden & Pionetti, 2011; Tafa, 2019). For this reason, formalization initiatives can face challenges gaining the acceptance and participation of members, especially in contexts where groups were initially built from the bottom up.

Existing literature does little to explore the process of formalization and, particularly, the role of trust. Most of the literature on the formalization of livelihood support focuses on the benefits and drawbacks of formal and informal economies ( Nelson & de Bruijn, 2005; Putzel et al., 2015; Xheneti et al., 2017). For example, research in Kenya demonstrated that the formalization of savings groups positively affected group financial performance ( Kirui & Onyuma, 2019). Meanwhile, anthropological work in Tanzania highlighted how formalization can affect in-group functioning by suggesting that stricter savings and loans rituals lead members to interact with the group in different ways ( Green, 2019). These studies highlight the way formalization shifts social dynamics within savings groups and supports greater economic benefits for participants, but they crucially overlook the formalization process itself and fail to highlight the trust landscape which underlies the success of formalized interventions.

In this paper, we address this important gap by examining the savings group formalization efforts of the Nigeria for Women Project (NFWP), a federal initiative to promote women’s livelihoods, economic empowerment, and gender equality in 32 states in Nigeria. (The project is currently scaled up across 32 states in Nigeria, but this study focuses on the initial pilot across six states.) Evidence from across the world, particularly from India and from sub-Saharan Africa, indicates that such groups, comprised primarily of women, can have positive effects on financial inclusion, asset ownership, women’s agency, food security, and resilience to economic shocks ( Adegbite et al., 2022; Barooah et al., 2020; Brody et al., 2017; de Hoop et al., 2022b; Desai et al., 2019; Gugerty et al., 2019; Gash & Odell, 2013). We focus particularly on the role of institutional trust within the formalization process. Using qualitative data collected in two waves in 2022 and 2023, we investigate how the NFWP encountered mistrust in its effort to formalize women’s groups, highlight strategies used by the NFWP to overcome community-level mistrust, and examine the success of these strategies and public perceptions of the project over time.

The study provides evidence for the initial causes of skepticism toward the project and present promising strategies for gaining community buy-in for women’s group formalization efforts in Nigeria. These findings may apply to other savings group formalization projects and community-based interventions in Nigeria and beyond, in cases where institutional mistrust poses a challenge to the implementation of crucial livelihood supports. In this way, the paper contributes to an emerging literature on the role of trust in the formation of savings groups in Africa and self-help groups in India (e.g., Jakimow and Kilby, 2006; Kumar et al., 2018; Nichols, 2021).

## Literature

Scholars and development professionals have long recognized the role of trust in facilitating (or impeding) community-based interventions. For instance, in the public health field, researchers have sought to understand how to improve the uptake and acceptance of vaccinations and epidemic response, among others ( ARK, 2018; Enria et al., 2021; Gupta et al., 2021; Limani et al., 2018; Mayhew et al., 2021; Tsai et al., 2017, 2020; Yang & Huang, 2021). Other studies have noted the importance of gaining community trust for institutionalized digital inclusion projects ( Madon et al., 2009). Within such studies, authors indicate that although some dynamics of trust are similar across cultures, many factors related to trust are highly contextual ( Kaasa & Andriani, 2022; Saunders et al., 2010). For instance, Saunders et al. (2010) asserted that respect for authority plays a more important role in collectivist cultures like Nigeria. Nichols (2021) found that long-standing savings and loan groups operating in economically stable areas in India exhibited higher levels of trust in implementing organizations than did more early-stage groups operating in areas with higher levels of poverty. To draw out the nuances of a given trust landscape, some academics have differentiated between “personalized” or interpersonal forms of trust and “civic or institutional” trust, a distinction that is also useful for understanding dynamics of formalization ( Aggarwal et al., 2015; Bachmann, 2018; Epstein & Yuthas, 2011; Sabhlok, 2011; Sztompka, 1999).

Regarding savings and loan groups, there is ample evidence to indicate that personalized, in-group trust affects their functioning and long-term sustainability. Because such groups rely on the cooperation of members, for example, to save and loan to one another, peer trust is vital to the smooth functioning of these groups ( Anderson et al., 2014; Molyneux et al., 2007; Nasir, 2021; Nawaz, 2017; Pradeep & Rakshitha, 2016). Research has highlighted how higher levels of trust between group members can improve loan repayment rates and strengthen the economic impact of savings groups ( Anderson et al., 2014; Hunter et al., 2020). High levels of personalized trust can also facilitate the impact of women’s savings groups on economic and women’s empowerment outcomes ( Epstein & Yuthas, 2011; Sabhlok, 2011; Satish, 2001). By contrast, low levels of in-group trust can undermine group success; issues like corruption, theft, and elite capture can fuel mistrust and contribute to the failure of some savings and loan groups ( Molyneux et al., 2007; de Cordier, 2009). While these studies indicate the relevance of personalized trust in savings group interventions, less is known about how *institutional* trust affects these groups. While institutional trust may be less relevant for informal savings and loan groups, it is of key importance to formalization initiatives such as the NFWP. The transition from informal to formal structures requires members to trust not only their personal relationships, but also the implementing institution ( de Hoop et al., 2022a). If institutional trust is weak, members may resist formalization due to fears of corruption, exclusion, or exploitation.

Within the literature on formalization, research indicates several challenges for a program such as the NFWP. For instance, Putzel et al. (2015) found that formalizing an already informal structure, such as existing savings and loan groups, introduces a risk of elite capture and can generate negative effects for women or other marginalized groups. Formalization can also limit access of the poor, women, and other marginalized populations who may have better access to the informal economy ( Nelson & de Bruijn, 2005; Xheneti et al., 2017). Despite challenges to formalization within the Nigerian context, research from Ilorin, Nigeria found women to be more open to formalization of savings groups than men ( Abdul-Yakeen & Gatawa, 2015), thus presenting an opportunity for the NFWP.

The NFWP is an initiative of the Government of Nigeria supported by a $500 million credit from the World Bank ($100 million at the time of this study). The program aims to establish new women’s saving and loan groups and transform existing informal women’s or mixed-gender groups into formal, women-only groups, called women’s affinity groups (WAGs). WAGs are formal savings groups that provide programming in four areas: the development of social capital, the building of livelihoods, the creation of partnerships, and messaging about gender and other social norms. The NFWP aims to improve women’s livelihood opportunities and facilitate women’s access to economic markets by mobilizing them into WAGs.

Since 2019, the Federal Government of Nigeria and the World Bank have implemented the NFWP across six states of Nigeria– Ogun, Taraba, Kebbi, Abia, Niger, and Akwa Ibom– with efforts underway to scale up the program to over 32 new states starting in 2024. NFWP implementation in the original six states has proceeded in several phases, beginning with WAG formation. Following the formation of new WAGs, the project extended a full package of training, including savings and credit group management and administration. In the subsequent phase, WAG members received training on personal finance, entrepreneurship, and business management. The NFWP also disbursed small grants to women who presented viable business plans. In addition, Livelihood Collectives (cottage industries) were formed by members of WAG across similar value chains supported with operating costs, equipment and technical assistance.

While there is little scholarly evidence on institutional trust toward savings group formalization interventions, NFWP implementers anticipated some challenges because, as project planners wrote, “the Nigerian landscape is replete with bad experiences of microfinance and defaults in savings and loans schemes” ( World Bank, 2018, p. 28). Indeed, literature on institutional trust in the Nigerian context has documented mistrust toward local and federal governments, primarily as it relates to elections and service delivery ( Aluko, 2017; Ahmad et al., 2015; David-West et al., 2021; Majekodunmi, 2012; Omotesho et al., 2015; Page & Wando, 2022; Salisu et al., 2023; Yahya, 2006). These authors broadly characterize Nigeria, especially its northern regions, as “low-trust settings” where “citizens believe authorities are predatory or malevolent” ( Tsai et al., 2020, p. 3). David-West et al. (2021) echoed this, writing of Nigeria’s financial sector, “there is a systemic distrust of government led initiatives … many of the unbanked poor would rather trust local schemes they can relate with” (p. 22). In their synthesis of the literature on trust, Saunders et al. (2010) noted that people often weigh evidence that erodes trust more heavily than evidence to the contrary, thus presenting an especially steep hurdle for the NFWP to overcome. Chukwuma et al. (2019) meanwhile examined strategies to build trust in public services in Nigeria and suggested that service providers who share identities related to “ethnicity and religious affiliation” are more likely to be trusted (p. 2). Together, these authors illustrate the importance of understanding the nuances of institutional trust in Nigeria and learning from the way that the NFWP encountered and navigated trust-related challenges.

## Methods

The findings in this paper are based on qualitative data collected in early 2022 and late 2023 as part of a mixed-methods impact evaluation of the NFWP. We employed three core qualitative data collection methods:

1) Focus group discussions (FGDs) with WAG members; former women’s group members and members of non-WAG women’s groups; and husbands of WAG members.
2) In-depth interviews (IDIs) with WAG members and women not participating in WAGs.
3) Key informant interviews (KIIs) with staff from the World Bank as well as Federal, State, local government area (LGA)-level, and ward level officials and project staff.

In the first wave of data collection, Akwa Ibom had not yet been selected for inclusion in the project, so it was omitted from data collection. In the second wave, we visited all six states. In both rounds, we purposively selected two LGAs and one ward per LGA. Of the two wards sampled in each state, one was an urban environment, and one was more rural. In total, we collected qualitative data from 10 wards across five states during the first data collection round, and we visited 12 wards across six states during the second data collection round. The qualitative sample for the first round of data collection comprised six FGDs and four IDIs with beneficiary respondents per ward, for a total of 30 FGDs and 20 IDIs. During the second round of data collection, we conducted one FGD with WAG members in each ward, and we alternated between IDIs with members and non-members, conducting six of each in the total sample. The number of KIIs differed between the two rounds as we included additional project staff during a second data collection round, for a total of 41 KIIs during the first round of data collection and 60 KIIs during the second round of data collection.  Table 1 summarizes the sampling of FGDs, IDIs, and KIIs across both rounds of data collection.

**Table 1.** Sampling of FGDs, IDIs, and KIIs (both rounds).

	FGDs		IDIs		KIIs	
	Round 1	Round 2	Round 1	Round 2	Round 1	Round 2
WAG members	10	12	10	6	**-**	**-**
Non-member women	10	-	10	6	**-**	**-**
Spouses of WAG members	10	-	-	-	**-**	**-**
World Bank staff	-	-	-	-	2	1
National-level NFWP staff	-	-	-	-	4	8
State-level NFWP staff	-	-	-	-	15	10
LGA-level NFWP staff	-	-	-	-	10	24
Ward-level NFWP staff	-	-	-	-	10	17
Total	**30**	**12**	**20**	**12**	**41**	**60**
Grand total	**42 FGDs**		**32 IDIs**		**101 KIIs**	

We prepared the data collection instruments in advance of each data collection mission, in collaboration with key project stakeholders. The questionnaires covered a wide range of topics relevant to the broader NFWP evaluation, including participant experiences with WAGs, perceptions of community gender norms, challenges and facilitators of NFWP implementation, and the perceived benefits and costs of WAGs. Next, we trained interviewers and piloted the research tools in a non-research site before conducting each field mission. In the field, data collection teams gained the informed consent of all participants before facilitating a semi-structured discussion based on the questionnaires. Data collectors recorded their conversations, which took place in English or one of the three major local languages in Nigeria (Hausa, Igbo or Yoruba). Using audio recordings, enumerators transcribed and translated the data into English.

We then analyzed the qualitative transcripts following an iterative process of coding, analysis, and summation. We developed a coding structure based on our interview protocols and deductively coded all transcripts. We also inductively added themes to the codebook as they arose in the data. We used thematic analysis to identify patterns, deviations, and major themes in the qualitative data. We further conducted sub-sample analyses to identify any thematic patterns across treatment areas, states, and regions as well as urban and rural areas. A subset of findings was reported in the baseline and midline reports for the evaluation of the NFWP ( de Hoop et al., 2022b; de Hoop et al., 2025), while we selected a more focused set of thematic findings for this paper.

This project was reviewed and approved by the AIR Institutional Review Board (IRB00000436), which is registered with the Office of Human Research Protection as a research institution (IORG0000260) and conducts research under its own Federalwide Assurance (FWA00003952). We also received IRB approval from the National Health Research Ethics Committee, Nigeria. We obtained written informed consent from all research participants.

## Findings

We organize our findings into three sections which roughly correlate to the time progression of the project. Saunders et al. (2010) offer a five-stage model for trust development, namely context, opening stance, early encounters, breakthrough or breakdown, and consequences. This analysis of the NFWP provides a case study of institutional trust development through the first three stages: First, we present findings on how community members perceived the NFWP upon its initial rollout. Next, we examine strategies the project used in the recruitment phase to overcome community mistrust. Finally, we highlight how trust transformed over time through the implementation of the project.

### Initial encounters with NFWP: Mistrust and fear

While we identified that some women did not enroll in the NFWP due to misinformation about the requirements for joining and their lack of income (e.g., to contribute their savings to WAGs), many interviewees suggested that feelings of mistrust kept them from joining WAGs. In all geographies where data collection took place, the research team encountered reports of hesitation, fear, and skepticism among members of the community toward WAGs as the NFWP was initially rolled out. Two key drivers of this mistrust emerged in the data: 1) community members’ negative past experiences with other community-based development initiatives; and 2) a broader context of mistrust toward the Nigerian government.

Most respondents who expressed feeling mistrustful of the NFWP cited the high frequency of fraudulent savings and livelihood interventions, which disappointed them in the past. Respondents throughout Nigeria, and in both rural and urban settings, described how numerous organizations entered their communities with the stated objective of helping poor women grow their income (see e.g., Alabi, 2019). Yet, most did not deliver on their promises. Together, the respondents broadly described this as a recurring trend whereby external organizations gained community members’ buy-in, took their money, and ultimately failed to deliver on their promises to support women’s livelihoods. As a WAG member in Niger state told us,

“[Other associations] come to tell us that they want to help women with money for business activities but that we have to pay money for it. Even when you don’t have the money, you will go and borrow to pay these fraudsters. But in the end, you will not hear from them anymore. You did not get the assistance, and your money has gone. That is why when [NFWP] came in, not all of us wanted to be part of it.”

Across our sample, many respondents had personal experiences with such failed schemes, but even those who were not directly affected described there being a broad distrust of NFWP throughout the community. A male community member detailed his experience of seeing failed initiatives come to his community:

“Different associations sprout up and then come to the community to collect money from the women…after paying and hoping that the next visit will be them getting the things they promised them, they will not hear from these people again. Meaning their money is gone and they still did not get the assistance that was promised.”

These fraudulent schemes had the same purported objectives as the NFWP. When we asked a respondent in Niger state how she was convinced to pay money to these earlier programs, she replied, “They come to tell us things that we want to hear. They will come to tell us that government wants to assist us because we are poor.” As a government-sponsored program aiming to promote women’s economic empowerment, the NFWP may have resembled those past projects for community members and therefore elicited mistrust from women and men in many communities.

Respondents commonly expressed that those individuals who found themselves defrauded by malicious organizations had no means for recourse. As one project staff reported, “after they gather the money, they will just run away and you will not see them.” The spouse of a WAG member described, “After paying … they will not hear from these people again, meaning their money is gone, and they still did not get the assistance that was promised.” The failed schemes appeared to be led by Nigeria-based organizations such as business groups, religious organizations, and local non-governmental organizations. Some respondents viewed the NFWP in the same light as these other schemes, seeing the project as a high-risk opportunity relative to existing community structures such as informal and local savings groups.

Regarding the association of the NFWP with the government, we encountered mixed reports of trust in local and federal governments. Sensitizations about the project underlined the fact that NFWP was a government initiative, but this fact both encouraged and discouraged trust in the NFWP, according to our respondents. For instance, some respondents reported that they *mistrusted* the NFWP due to its association with the government. The World Bank (2018) planning document for the NFWP foresaw this reaction within target communities, noting, “there is limited confidence among women on the ability of the (remote) local government to support their economic activities” (p. 27). One WAG member in Abia state confirmed this, saying, “Some found it very hard to trust [the NFWP] … they said the government was out to scam us.” Other respondents reported their fears that the government would embezzle project funds, and several said that they had little confidence in the government because it had overlooked their communities when it came to providing other public services. Some participants made comments like, “the government does not care about the conditions of the roads here” (Spouse of women’s group participant, Abia) or that the government only supports rich and privileged women (Women’s group members, Taraba). Speaking about the NFWP, one woman in Taraba state who did not join WAGs expressed her doubt: “Among government-based associations, I have not seen any successful one.”

On the other hand, and in equal proportion to this skepticism, we received reports that the NFWP’s affiliation with the government *engendered trust* by distinguishing it from the fraudulent programs that had come to communities in years past. Such respondents expressed the belief that the government had the capacity and responsibility to support them. As one WAG member attested, “That was what attracted us, that the government will help us.” A WAG member’s spouse echoed this: “We asked those organizing it and they assured us that government will help them. That’s why we say they should continue.” In the eyes of these respondents, the project’s association with the federal government lent it credibility that past, fraudulent initiatives lacked. Such respondents indicated a strong sense of optimism and expressed that government assistance would be crucial to helping them escape poverty. Focus group participants made comments like, “God shouldn’t make us suffer. We can only rely on government to help us” (female community member in Ogun), and “There is nothing we can do. It’s the government […] we are begging for” (spouse of a WAG member in Ogun). Overall, the project’s association with the government had mixed results on prospective participants, with some feeling discouraged and others feeling encouraged to join.

The mistrust we observed had clear effects on the NFWP and the uptake of WAGs in targeted communities by preventing a proportion of women from joining the project. Among those who chose not to join a WAG, many described adopting a “wait and see” approach by deciding to observe the WAGs and deferring their decision for a later time. This suggests that the women lacked confidence and sufficient information to feel comfortable joining at that time. Indeed, those who joined broadly expressed signing up for the project despite their mistrust, and they said things like “we joined with fear but now we are enjoying it.”

Further, beyond a woman’s own attitudes toward the project, mistrust in the NFWP sometimes affected her husband’s support for the project. Across our study, respondents shared that households typically abide by traditional gender roles within the Nigerian context, both in regard to household responsibilities and decision-making: Women are expected to manage household chores, take care of the children, and obey the husband, while men are considered the “head of the household,” charged with household leadership and meeting financial needs. Although many people highlighted that some husbands and wives make joint decisions, particularly on issues related to children, most said that husbands have the final say when it comes to household decisions about healthcare, education, etc. A husband’s permission was therefore necessary to join a WAG, and like women, many men were also skeptical of the NFWP. A woman in Kebbi state described,

“I told him [my husband] about the association. He started saying ‘Are we not tired of all these associations?’ or ‘How long will they keep deceiving us?’ And that I should not join. Later I went back to him and asked him to just let me try, and he permitted me to join.”

As this woman described, her husband was eventually convinced to allow her to join. However, another, smaller group of women were ultimately prevented from joining a WAG because of their husband’s mistrust and disapproval.

### NFWP strategies to generate trust

The NFWP’s core planning document anticipated potential mistrust even before the project launch, leading the project to employ several strategies which ultimately resulted in the successful recruitment of over 450,000 women into nearly 22,000 WAGs during the initial pilot ( World Bank, 2023). Interviews and focus group discussions in NFWP communities indicated that community mobilization efforts, the formality of the WAG model, transparency of WAG procedures and policies, use of project champions, the consistent presence of the NFWP, the positive outcomes members experienced through WAGs, and the NFWP’s affiliation with the government helped build trust in the NFWP and facilitated women’s participation in the WAGs.

Regarding community mobilization, many WAG participants indicated that the sensitization efforts of the NFWP were a crucial part of their decision to join the project. Among other efforts such as radio messaging and announcements at local markets– activities guided by the project’s central communications strategy– project implementers also approached traditional and religious leaders to serve as “champions” of the NFWP. These champions then helped advocate for the program among community members, particularly male heads of household, whose permission and support were generally important determinants of whether a woman could participate in a WAG or not. Community-based champions continued to support WAGs even after this sensitization phase, sometimes providing a space for WAG meetings. We found that inter-household dynamics as well as social and cultural norms varied by region. For example, in the northern states (e.g., Taraba, Kebbi, Niger), women had relatively fewer opportunities to socialize outside the home other than through informal networks and social ceremonies. In these areas, program implementers targeted these informal networks and social ceremonies to find local leaders who were willing to support WAGs. By contrast, in southern Nigeria (e.g., Abia, Ogun), the program could approach women through more formal networks– for example, through gatherings hosted by community leaders or existing women’s groups. In both cases, the participation of local leaders and respected community members signaled the project’s credibility. As one WAG member explained, “When [the NFWP] came, I asked who was in charge and they told me Mama Adekoya. My mind was at rest when I saw respectable members of the society.” By tapping into local community networks– both formal and informal– project implementers were able to tie the NFWP to the legitimacy and credibility of respected figures in the community, thereby generating trust for WAGs and the project overall.

*In addition, * study participants indicated that the structure of the WAG model helped to portray the NFWP as transparent and accountable. The WAG model, developed by the government and World Bank, involves established principles, rules, and penalties to regulate the collection of money, member attendance and tardiness, and loan default. The NFWP used a cascade approach to convey the WAG structure: NFWP facilitators first met with community leaders to obtain their official support and, subsequently, held general awareness meetings with men and women in the community. Through sensitization about the unique rules of WAGs, respondents came to see the NFWP as distinct from other less formal savings groups in that they had an established structure. These meetings clearly laid out the rules of WAGs, including that WAGs would elect their own leaders, develop their own bylaws, keep their own savings in a box with three locks (i.e., with keys held by three different WAG members), and would face no hidden fees from the NFWP. As a WAG member summarized, “When this association started, we thought it will be like the former ones that came and folded up…The difference [with NFWP] is that there are rules and regulations governing the group.” NFWP facilitators and community leaders also emphasized that savings would be left with the community (in the three-lock box) and not held by the NFWP. The WAG savings-box model particularly facilitated the perception of WAGs as transparent. As one woman noted, “they [the WAG leaders] have been given the boxes and the keys; they are all in their hands. […] The association is very transparent.” Respondents noted feeling that their money was safe and would be used for its intended purpose, helping to assuage their concern that the organization would steal their money, as others had done in the past. Thus, the structures, regulations, and defined processes– paired with extensive, community-based sensitization at the recruitment phase– suggested the formality of the WAG model and engendered more trust in the program.

### Trust *Generation through* NFWP implementation

At both the first and second round of data collection, we found that community members’ observations of NFWP implementation strengthened their confidence and trust in the project, enticing many who had previously distrusted the project to consider joining. At the time of our first wave of data collection, the project had been active for around 14 months. Respondents had already observed the project’s consistent presence in the community and successful savings and loan activities. This consistency addressed communities’ concern– based on past experience– that the project would disappear without warning and led them to view the project as accountable and reliable. For instance, some respondents cited that they eventually came to see the value of the NFWP after observing its savings and loan activities and trainings provided to WAGs in their community. In addition, respondents mentioned the ongoing WAG meetings and regular presence of project staff as signs that the community could rely on the project to provide support in the long term. The project’s on-ground presence did much to dispel fears that the NFWP would disappear, abandoning women like other organizations had done in the past. While the project also established a formal grievance redress mechanism, women rather cited the possibility of informally approaching a project staff as a sign that they could air their complaints.

While the NFWP had gained many people’s trust through consistent presence in the communities, we found that the sustained credibility of the project still depended upon its successful dispersal of individual livelihood grants, which had not yet occurred at the time of the first round of data collection. These small grants were a much anticipated element of the program and many WAG members, their spouses, and even project staff indicated during the first round of data collection that their continued trust depended upon the project following through as promised with the delivery of these grants. As the husband of a WAG member in Kebbi claimed, “Every woman in this association has the hope that the government will help her at the end of the project with money to continue her business. If this hope is broken, they will never trust any association that comes again.”

By the second wave of data collection, livelihood grants ranging in value from N30,000 to N60,000 (US$68 to US$135) had been distributed to a total of 235,003 WAG members ( World Bank, 2023). At that time, we observed strong levels of trust in the project and in the government attributed to the distribution of livelihood grants to WAG members. As a grantee in Ogun state described, community members’ mistrust was transformed through the grant distribution process:

“With time, everybody got the grants. There have been many forms that we filled out before now that we didn't hear anything about, but this one is different because we heard from them, and we received our Grant. All the people that said it's a lie started to believe that it is true.”

Other respondents, too, indicated that the successful delivery of this promised element was a catalyst for building the credibility of the project. According to one project staff member in Ogun state, the grant distribution was the moment the project became “real” for NFWP communities: “People did not trust it, but after testing it and they found out that it was real, we did not face any problems again.” This trust further appeared to extend to the government itself, with women telling us that they had come to trust the government due to the results they observed in the NFWP. For example, a WAG member in Taraba state explained, “The federal government is trustworthy because they promised us grant and the grant got to us without hindrance.” Although trust in the government was mixed during the first round of data collection, results from the second data collection round indicated high levels of trust in the government and project in NFWP communities. The provision of livelihood grants through NFWP clearly demonstrated to recipients and their families that the institution can deliver promised services.

As trust in the project grew over this time, some respondents noted that many more people rushed to sign up for WAGs. Despite this, many women during the second round of data collection told us that they had not seen an opportunity to join after the initial group formation period. Indeed, after initial enrolment (i.e., before our first round of data collection), the NFWP halted recruitment of new members to assess the capacity of ward facilitators to oversee existing WAGs. This process took upwards of six months and did not always result in the formation of additional WAGs. Thus, in trying to mitigate the risk they perceived in joining the NFWP, some women missed their opportunity to participate in the project.

## Discussion & Conclusion

The evidence presented in this paper illustrates how practitioners who aim to implement savings groups or comparable women’s self-help groups must account for the broader context of institutional trust and mistrust in the areas in which savings and loan groups operate– ideally during the recruitment and group formation phase. For projects such as the NFWP, which operates in a low-trust context and relies heavily on community members’ willingness to form WAGs, institutional trust proved to be both a key hurdle and opportunity to successfully implement the project. Our paper highlights how institutional mistrust can undermine savings group formalization initiatives, particularly in the earlier stages of trust formation within groups as outlined by Saunders et al. (2010): the initial context, the opening stance to sensitize potential members, and the early experiences with the program. The findings suggest that understanding the complex interplay between personalized trust inherent in informal women's groups and the institutional trust required for formalized groups was crucial in the development of NFWP strategies to generate trust.

For the NFWP, the primary driver of community members’ initial mistrust was negative past experiences with other livelihood interventions including other savings and loan group programs and microfinance initiatives. Although such negative experiences can be especially difficult to overcome as related to trust ( Saunders et al., 2010), NFWP implementers employed several strategies to generate trust in a way that addressed the key drivers of institutional mistrust including community mobilization, transparent processes, and consistent implementation. Consistent with existing literature showing that experiences with service provision significantly shape citizens’ views of the government ( Kaasa & Andriani, 2022; Lavallée et al., 2008), we found that individual perspectives on the government shaped attitudes toward NFWP both by building trust and sowing fear in the project. Although we are not alone in observing the lack of trust toward government actors in Nigeria ( Majekodunmi, 2012; Page & Wando, 2022; Salisu et al., 2023; Yahya, 2006), our data indicated that the NFWP’s affiliation with the government legitimized the program and encouraged participation for some women. From these findings, we identified three key implications to guide implementers in their approach to designing the formation phase of formalized savings group projects.

First, conducting community mobilization prior to implementation allows an initiative to cultivate the buy-in and community ownership from key leaders and champions. In the Nigerian context, formalizing women’s groups required external mediation such as the engagement of respected local leaders to balance personalized trust with institutional trust during transition. While the NFWP employed multiple awareness-raising strategies for the program such as radio messaging and announcements at the local markets, the engagement of influential religious and community leaders most saliently contributed to building trust among community members, who often noted the importance of these champions in shaping their views of the program. This strategy of first winning trust of respected community members aligns with literature indicating that collectivist cultures like Nigeria particularly value the views of authority figures in trust-generating processes ( Saunders et al., 2010), as well as examples of trust-building within the formalization process of other types of development initiatives ( Madon et al., 2009).

Second, providing multiple opportunities for people to join over time allows implementers to show early positive outcomes from participating and to prove that the project intends to stay over the long term. Sabhlok (2011) terms this the “demonstration effect,” or the fact that examples of a well-performing project can encourage non-participants to join later. In the NFWP, some women reported that they waited to observe the WAGs before joining, a delay which allowed them to see the project’s positive results among those who joined in the first recruitment wave. Importantly, this method of mobilizing trust only matters if the project design, particularly at the recruitment phase, allows for participants to join once the demonstration effect is achieved. A project or program can do this by designing a pilot phase or by planning multiple waves of recruitment.

Third, by designing and providing guidelines and conducting regular training, project implementers can support the formalization process, which has been shown to address some of the underlying drivers of institutional mistrust ( Xheneti et al., 2017). In the NFWP, formal mechanisms established a regularity and predictability in the WAG’s operation that helped establish expectations among the women participants. The NFWP also created a commonly shared and transparent reference point that group members could reliably turn to if somehow their expectations were not met. This reference point helped address underlying concerns about the lack of recourse for women who had negative past experiences with similar projects.

And lastly, by understanding the general context of trust or mistrust in government-affiliated institutions, project implementers can anticipate how much they might want to present government involvement in the formation phase. As reported above, the NFWP’s affiliation with the government was generally understood as a liability in some areas, whereas in others, it was considered an asset. With this context in mind, if project implementers in these latter areas partnered with recognizable local government actors to lend their legitimacy, credibility, and visibility to WAGs, it would have been reasonable to find a positive influence on group formation. On the other hand, perhaps less visibility of government partners may be warranted in areas that have a low level of trust toward government institutions.

Regardless of if the broader institutional context fuels trust or mistrust in a savings group project, our paper found that the NFWP itself was an opportunity to shape individual and community’s perceptions and levels of trust in the surrounding institutions. As aligned with existing literature showing that the quality of public service delivery and the involvement that the state takes in its citizens’ interests can significantly affect people’s views of the government ( Kaasa & Andriani, 2022; Lavallée et al., 2008), we found that after the NFWP demonstrated its value to communities, community members came to view the project positively, and by extension, the government gained credibility through its affiliation with the project. Thus, we found that institutional trust not only shapes people’s participation in formal savings and loan groups, but participation in the formalized groups may also influence people’s institutional trust.

## Data Availability

This paper relies on qualitative data owned by World Bank Nigeria. The data can be accessed by reaching out to authors Yetunde Fagotun ( yfatogun@worldbank.org) or Michael Gboyega Ilesanmi ( milesanmi@worldbank.org).
